# The Utility of Biliary Manometry in Assessing Early Catheter Removal After Percutaneous Balloon Dilatation of Hepaticojejunostomy Strictures

**DOI:** 10.7759/cureus.22761

**Published:** 2022-03-02

**Authors:** Sunil Kumar, Selvamurugan Vignesh, Deb K Boruah, Archna Gupta, Rajanikant R Yadav, Vinay Kumar Kapoor, Anu Behari, Supriya Sharma

**Affiliations:** 1 Department of Radiodiagnosis, Sanjay Gandhi Postgraduate Institute of Medical Sciences, Lucknow, IND; 2 Department of Interventional Radiology, Meenakshi Mission Hospital, Madurai, IND; 3 Department of Radiodiagnosis, Lakhimpur Medical College, Lakhimpur, IND; 4 Department of Surgical Gastroenterology, Mahatma Gandhi Medical College & Hospital, Jaipur, IND; 5 Department of Surgical Gastroenterology, Sanjay Gandhi Postgraduate Institute of Medical Sciences, Lucknow, IND

**Keywords:** internal external biliary catheter, biliary manometry, percutaneous balloon dilatation, iatrogenic bile duct strictures, percutaneous transhepatic biliary drainage, ptbd, roux-en-y hepaticojejunostomy

## Abstract

Background and objective

Percutaneous balloon dilatation followed by long-term internal-external biliary catheter (IEBC) placement is the standard radiological management for postoperative hepaticojejunostomy (HJ) strictures. The treatment is considered successful when cholangiography shows a free flow of contrast across the anastomosis and the patient passes a "clinical test". However, these tests may not be suitable predictors of long-term successful treatment outcomes. The purpose of this study was to assess the utility of biliary manometry in the evaluation of successful treatment outcomes after HJ stricture dilatation and IEBC placement and its efficacy as a tool for early catheter removal.

Patients and methods

A total of 14 patients underwent percutaneous balloon dilatation of HJ strictures with IEBC placement. A two-to-three-month interval was maintained between sessions of exchanging or upsizing IEBCs. Biliary manometry was performed after a mean duration of 6.3 months. Intra-biliary pressure of <15 mmHg was considered as the success threshold.

Results

Among the 14 patients, 11 patients passed initial manometry and had their IEBCs removed and were followed up for a mean duration of 47.8 months. Of these, one patient developed biliary obstruction after six months and underwent repeat HJ stricture dilatation and long-term IEBC placement. Three patients failed manometry and underwent re-dilatation of HJ strictures with IEBC placement. Using Kaplan-Meier survival analysis, the probability of patients remaining stricture-free after HJ stricture dilatation was found to be 100% at three months and 91% at six, 12, 18, 24, 36, and 47.8 months.

Conclusion

Biliary manometry prevents subjective variations in determining treatment endpoints and helps to assess early catheter removal after percutaneous balloon dilatation of HJ strictures.

## Introduction

Roux-en-Y hepaticojejunostomy (HJ) is currently considered to be the definitive treatment for iatrogenic bile duct strictures [1]. The incidence of HJ stricture following HJ is 2.6-17% as per various studies [2-4]. The treatment options available for HJ strictures include either surgery or percutaneous radiological intervention. Percutaneous management includes percutaneous transhepatic biliary drainage (PTBD) followed by balloon dilatation of the HJ stricture(s) [5-7]. The anastomotic site is kept patent by the placement of internal-external biliary catheters (IEBCs), which have varied in caliber from 8 to 18 Fr in different studies [8-11]. Thereafter, repeat cholangiography with or without balloon dilatation along with catheter exchange/upsizing is done at intervals of weeks to months (one week to three months in different studies) [8-9,11-12]. These biliary catheters have been placed for varying periods (mean duration of 1.1-19.9 months) in previous studies [13-14]. The endpoint of the treatment is considered to be reached when cholangiography shows the free flow of contrast across the HJ anastomosis into the jejunum and the patient passes a "clinical test" [8,15].

The "clinical test" consists of the placement of a thin non-functional safety catheter without side holes above or across the stricture site for a period of 7-10 days. The patient is followed up with liver function tests (LFTs). If the LFTs are not elevated (total bilirubin: <1.3 mg/dl, conjugated bilirubin: <0.4 mg/dl, alkaline phosphatase: <150 U/L) and the obstructive symptoms do not recur, the treatment is considered a success and the catheter is withdrawn. If the LFTs are deranged, then either repeat balloon dilatation is performed with placement of IEBCs or the patient is referred for surgical management [8,15-16].

Biliary manometry is an objective test that helps to avoid subjective variations in the assessment of the patency of the HJ anastomosis [17]. The aim of this study was to assess the utility of the biliary manometric perfusion test in the evaluation of successful treatment outcomes after HJ stricture dilatation and IEBC placement and to examine whether it can be used as a tool for assessing early catheter removal after HJ stricture dilatation.

## Materials and methods

Patients

In this study, 14 patients (five males, nine females) who underwent HJ stricture dilatation during the period from October 2017 to January 2022 were retrospectively evaluated. The study was approved by the Institutional Ethics Committee at the Sanjay Gandhi Postgraduate Institute of Medical Sciences, Lucknow (IEC approval no: 2019-131-IP-EXP-10). The indication for HJ in these patients was an iatrogenic injury to the bile ducts that occurred post-cholecystectomy in 11 patients, post-choledochal cyst excision in two patients, and post-Whipple’s procedure in one patient. All patients presented with complaints of obstructive jaundice and cholangitis. Pre-intervention imaging was done in the form of either ultrasonography, magnetic resonance cholangiopancreatography (MRCP), or multidetector computed tomography (MDCT). Written informed consent was obtained from all patients prior to interventional radiological procedures detailed below.

All patients initially underwent one to three PTBD procedures via the segment III, VI, and/or V/VIII ducts. This was followed by HJ stricture balloon dilatation, which was done either in the same setting or after the resolution of cholangitis. Balloon dilatation was performed over an AUS guidewire (Cook Inc., Bloomington, IN), using balloon catheters (Mustang, Boston Scientific, Natick, MA) of 14-mm diameter for common hepatic duct (CHD) strictures and 10-mm diameter for individual ducts. The balloon catheters were inflated with sufficient pressure to fracture the stricture(s) and kept inflated for five minutes. Post-dilatation, 10-F IEBCs (biliary duct drainage catheter, Devon Innovations, Bengaluru, India) were placed via each biliary access with a plan to provide at least 30-F initial diameter across the HJ site in case of common anastomosis (jejunal anastomosis with CHD) where possible and at least 10-F initial diameter in case of individual duct anastomose. One patient had one IEBC, three patients had two IEBCs, and 10 patients had three IEBCs placed. The patients were followed up after two to three months with cholangiography. If there was no evidence of obstruction with uninterrupted contrast flow across the anastomosis, the biliary manometric test was performed provided at least three months had passed since the first insertion of IEBCs. If there was evidence of anastomotic narrowing, re-dilatation of the HJ stricture and either exchange or upsizing of IEBCs to 12-14 F was done, and follow-up after two to three months was advised.

Biliary manometric perfusion test method

**Figure 1:**
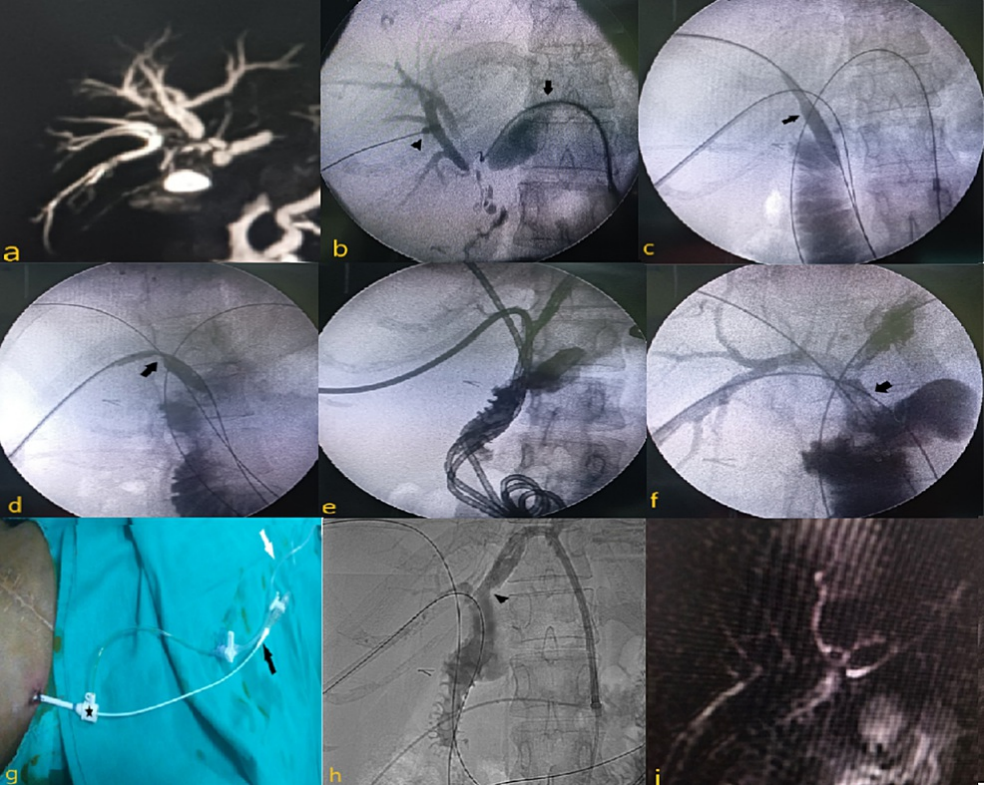
Details of the procedure in a 32-year-old female The patient presented with complaints of obstructive jaundice and cholangitis, two years after undergoing HJ for benign bile duct stricture. Her MRCP (a) showed dilatations of intrahepatic biliary radicles with stricture at the HJ anastomotic site of right anterior, right posterior, and left-side bile ducts. Percutaneous cholangiograms (b) via segment III biliary access (black arrow) and via right side bile duct puncture (black arrowhead) showed severe narrowing at the HJ anastomotic site with the minimal passage of contrast into the jejunum. Subsequently, 10-mm balloon dilations (black arrow) were done via right-side biliary accesses (segments VIII and VI respectively) (c, d) and also via the left-side segment III biliary access. This was followed by the placement of internal-external biliary drainage catheters (e) across the HJ anastomotic site. Cholangiograms (f) taken after three months of internal-external biliary drainage catheter placement showed good flow across the HJ anastomotic site (black arrow). Biliary manometry (g, h) was performed by placing an access sheath (*) in the segment VIII, segment VI, and segment III bile ducts after removing the internal-external biliary drainage catheters from the same ducts over a guidewire. A KMP catheter introduced into the sheath was connected to the manometer (black arrow) and its tip (arrowhead) was kept just proximal to the HJ anastomotic site. An infusion pump for the infusion of contrast was connected to the side port of the sheath (white arrow). MRCP (i) done three months after the manometry test showed no significant dilatation of intrahepatic biliary radicles HJ: hepaticojejunostomy; MRCP: magnetic resonance cholangiopancreatography

Biliary manometry was performed in a similar fashion to the technique reported by Savader et al. [17]. The IEBCs were exchanged for a 10-F vascular access sheath (Input Introducer Sheath, Medtronic Vascular, Minneapolis, MN) over a 0.038-inch Terumo guidewire (Radifocus, Terumo, Tokyo, Japan) and the sheaths were kept within the biliary ducts. After cholangiography through the sheath, a 5-F, 40-cm KMP catheter (Cook Inc.) was inserted through the vascular sheath and its tip was kept just proximal to the anastomotic site. The end of the KMP catheter was connected via a tubing and three-way stopcock to the manometer (Figure 1).

The side port of the vascular sheath was connected via another tubing and stopcock to the infusion pump. The tubing was flushed to remove any air within, and the baseline was zeroed with a manometer at the level of the mid-axillary line/heart. With the stopcock off towards the exterior and on towards the manometer, the baseline reading of the biliary system was recorded. If the pressure was <15 mmHg (<20 cm H_2_O), the procedure was continued. The infusion pump was then loaded with 50 ml of 50% diluted non-ionic iodinated contrast (Ultravist, Bayer Healthcare, Whippany, NJ). The biliary system was injected with contrast through the vascular sheath at the following rates: 2 ml/minute (120 ml/hour) x five minutes, 4 ml/minute (240 ml/hour) x five minutes, 8 ml/minute (480 ml/hour) x five minutes, 15 ml/minute (900 ml/hour) x three minutes, and 20 ml/minute (1,200 ml/hour) x two minutes. During each of these perfusions, the intra-biliary pressure was recorded using a biliary manometry set-up. The passage of the contrast and the position of the vascular sheath and the KMP tip were monitored fluoroscopically. In the case of patients having common anastomosis (jejunal anastomosis with CHD), contrast infusion and manometric reading were done in any one ductal system. If the patients had separate anastomoses for the right anterior, right posterior, and left ductal systems, then the biliary manometry test was performed individually for all these ductal systems. If at any point during the infusion the pressure within the biliary ducts exceeded 15 mmHg or 20 cm H_2_O or if the patient experienced abdominal discomfort/pain, the procedure was terminated and considered a failure. If the pressure remained below the cut-off level, the manometry test was considered a success. The patients were discharged with the placement of 5-F infant feeding tubes (IFTs) (Romsons Scientific & Surgical Pvt. Ltd., Agra, India) across the HJ site with end clamped and reviewed after one week with LFTs. The IFTs were removed if LFTs were not elevated. The patients were then followed up for any signs of biliary obstruction and with LFTs at one, three, and six months and then every six months thereafter.

## Results

**Figure 2:**
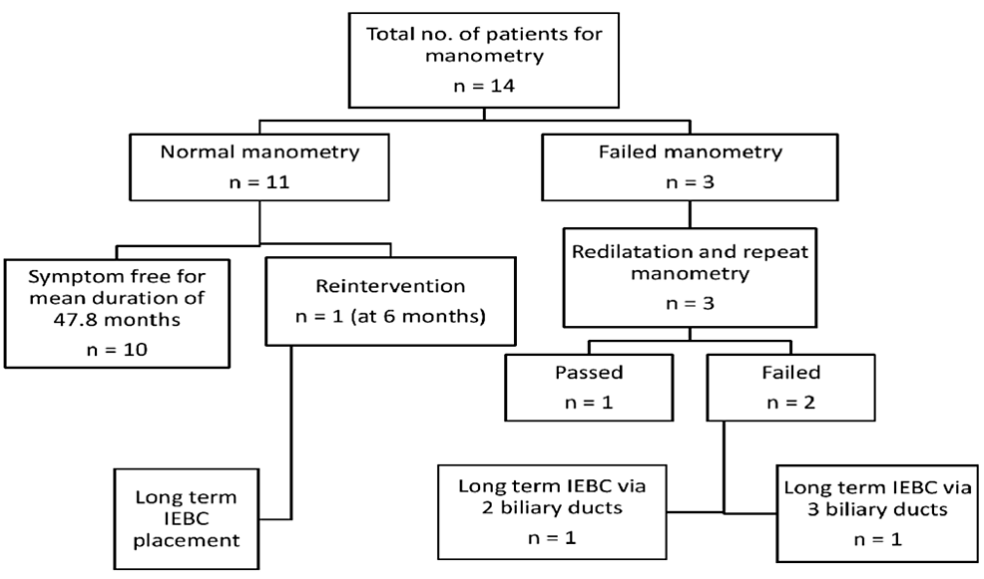
Flow chart showing the results of our study IEBC: internal-external biliary catheter

All 14 patients underwent percutaneous balloon dilatation for HJ strictures. Successful dilatation was achieved in all patients. An average of three dilatations (range: two to six) per patient was done. A biliary manometric perfusion test was performed in all patients after a mean interval of 6.3 months (range: 3-11 months) after the initial HJ dilatation and placement of IEBCs. The manometric test was done in all patients without any complications. Out of 14 patients, 11 patients (78.6%) passed the initial manometry test with their biliary pressures being <15 mmHg. Their drains were subsequently removed, and they were followed up clinically and with LFTs at one, three, and six months and then at six-monthly intervals. One out of these 11 patients presented with symptoms of biliary obstruction and elevated LFTs after six months. He underwent re-dilatation of HJ stricture and is on a long-term placement of IEBCs (Figure 2).

Three patients (21.4%) failed the initial manometry test with two of them having elevated baseline pressure (>15 mmHg) and one patient developing increased biliary pressure during the infusion. All patients who failed the initial manometric test underwent re-HJ stricture dilatation. They were placed on IEBCs and were followed up with manometry again at three months. On repeat manometry, one patient passed the test and had all three of his IEBCs removed, and was kept on clinical follow-up. One patient who was on three IEBCs for three separate anastomoses passed the manometric test via two ducts and had two IEBCs removed after passing the "clinical test". This patient failed the test via the segment III biliary access and underwent repeat balloon dilatation of the HJ stricture via the left hepatic duct and was placed on long-term IEBC via segment III only. One patient failed the manometric test via all three catheters on the second occasion also and was subjected to repeat balloon dilatation of the strictures and placed on long-term IEBCs. Out of four patients who failed the initial (n=3) or secondary manometry test (n=1), two patients (50%) passed subsequent manometry tests and had their IEBCs removed.

The mean follow-up period for patients who had initial normal manometric values was 47.8 months (range: 43.2-51 months). The mean duration of IEBC placement for these patients was six months (range: three to eight months). The mean follow-up period for patients who had failed initial biliary manometry was 47.9 months. Among them, the one patient who passed the repeat manometry had a follow-up period of 46.2 months, during which he was symptom-free. The remaining two patients are still on IEBCs. Details of patient management are listed in Table 1.

**Table 1:** Demographic and manometric data of patients IEBC: internal-external biliary catheter

Sr. No.	Age (years)	Sex	No. of IEBCs	Duration of IEBCs (months)	Initial manometry	Re-intervention after manometry	Repeat manometry after balloon dilatation and 3 months of IEBC placement	Follow-up duration after initial manometry (months)	Current status
1	60	M	2	9	Failed		Passed via the right side bile duct and failed via segment III duct	51.2	Long-term IEBC placement
2	29	F	3	3	Passed			51.0	Stricture-free
3	62	F	2	7	Passed			49.3	Stricture-free
4	65	M	3	7	Failed		Passed	49.3	Stricture-free
5	55	M	3	3	Passed			49.2	Stricture-free
6	55	M	3	9	Passed			48.4	Stricture-free
7	40	F	3	7	Passed			48.4	Stricture-free
8	60	M	3	4	Passed	Recurrence of stricture at 6 months	Passed	48.4	Stricture-free
9	49	F	3	8	Passed			48.2	Stricture-free
10	30	F	2	11	Passed			48.1	Stricture-free
11	32	F	1	3	Passed			46.5	Stricture-free
12	65	F	3	3	Passed			44.9	Stricture-free
13	58	F	3	4	Passed			43.2	Stricture-free
14	31	F	3	11	Failed		Passed via the right side bile ducts and failed via segment III duct	43.3	Long-term IEBC placement

**Figure 3:**
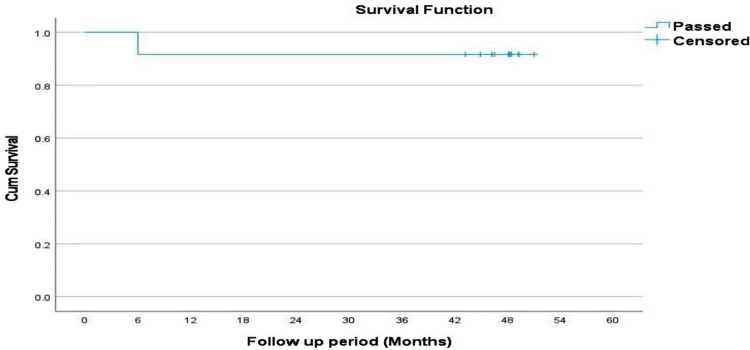
Kaplan-Meier survival analysis curve for long-term biliary patency in cases that passed the initial manometry test in our study

Using the Kaplan-Meier survival curve analysis, the probability of patients remaining stricture-free after HJ stricture dilatation was computed to be 100% at three months and 91% at six, 12, 18, 24, 36, and 47.8 months (Figure 3).

## Discussion

A major concern with the percutaneous management of HJ strictures lies in determining the duration of placement/timing of removal of IEBCs. This decision has largely been based on the subjective appearance of the free flow of contrast material across the anastomosis and the results of the "clinical test" [9-11]. The percutaneous procedure is considered a failure if there is a persistence of stricture even after three sessions of dilatations according to some studies [9-10,12,18].

The need for an objective assessment of the patency of the anastomosis and measurement of functional parameters has been proposed by some authors [13,19-20]. The long-term outcome of the dilated strictures depends mainly on the functional status, such as pressure and flow across the anastomoses. The pressure inside the ductal system is directly proportional to the degree of obstruction. Biliary manometry has been used for evaluating the outcomes of biliary surgery, endoscopic sphincterotomy, and other biliary conditions as a pre and postoperative tool [21-23]. If the pressure inside the biliary system is <20 cm H_2_O (<15 mmHg), it is presumed that the anastomotic site can withstand the normal flow of bile [17].

Previous studies on the biliary manometric test have clearly shown that manometry is as effective as the "clinical test" in determining the long-term patency of the HJ strictures [17,20,24]. Biliary manometry offers several advantages over the "clinical test" and direct cholangiography visualization. It is simple and safe to perform, and the entire procedure can be completed within one day. The "clinical test" requires a minimum of seven days to obtain results. The manometric test is based on the actual measurement of biliary pressures as opposed to the "clinical test" where LFTs are monitored. LFTs may be deranged in other conditions apart from the biliary obstruction, and hence may not always correlate with the degree of the obstruction. Biliary manometry plays an important role especially in patients with separate anastomoses for two or three ductal systems, where the specific ductal system that is stenosed can be identified, as seen in one of our patients. The procedure failures can be rectified with repeat balloon dilatation and maintained on follow-up.

On analyzing our results of manometry on 14 patients, 11 patients who passed the initial manometry had a mean follow-up period of 47.8 months. One patient who failed the initial manometry but was normal on repeat manometry done after three months was also symptom-free for a duration of 46.2 months. Using Kaplan Meier statistics, the long term biliary-enteric anastomosis patency in our group of patients was 91% at the end of one, two, three, and four years, which is comparable to the other studies that also show long-term patency rates ranging from 77-90% (Table 2).

**Table 2:** Comparison between our study and other studies on biliary manometry BBS: benign biliary strictures; HJ: hepaticojejunostomy

Variables	Köcher et al., 2011 [24]	Thomas et al., 2009 [15]	Haskal et al., 2008 [19]	Our study (2022)
Number of patients	30	12	10	14
Clinical indication	BBS post-HJ	BBS post-HJ	Liver transplants	BBS post-HJ
Mean duration of placement of biliary catheters		6.8		6.3
Passed initial manometry	28	11	10	11
Mean follow-up duration (months) of patients who passed initial manometry		15.3	8.4	47.8
Re-intervention in patients who passed initial manometry during follow-up	3	2	1	1
Long-term biliary patency in patients who passed initial manometry test	82.2% at 3 years	90.9% at 9 months, 77% at 1, 2, and 3 years	90% at 3, 6, and 12 months	91% at 1, 2, 3, and 4 years

One of the main concerns of the percutaneous management of anastomotic biliary strictures is related to the duration of IEBC placement after the dilatation of HJ strictures. Long-term placement of these catheters can be a source of physical and psychological discomfort to these patients. Pneumatic dilatation of the stricture causes injury to the anastomotic site, resulting in scar formation. The placement of the IEBCs keeps the stricture site open for a prolonged period, allowing scar remodeling and consolidation [25]. Remodeling is known to occur within six weeks to three months [26]. Therefore, we felt that the biliary manometric perfusion test can be done as early as three months post-HJ stricture dilatation, and patients can be offered early catheter removal if they pass the biliary manometric perfusion test without compromising the patency of the anastomotic site. In four patients in our study group, the manometry test was performed as early as three months after the placement of biliary catheters. All four patients passed the manometry test, and their catheters were removed and they were stricture-free for a mean duration of 47.8 months. Thus, apart from evaluating the success of the balloon dilatation, the manometric test can also be utilized for assessing the early removal of IEBCs.

Our study is limited by its small sample size and the relatively short duration of follow-up after manometric evaluation. Biliary strictures are known to recur even after 20 years of successful balloon dilatation [27]. Therefore, these patients need to be followed up for a long period of time. However, the promising results obtained in our study suggest that the biliary manometric perfusion could be used as a routine practice for assessing the time of IEBC removal after HJ stricture dilatation.

## Conclusions

Biliary manometry is an effective test in the evaluation of percutaneous balloon dilatation of HJ strictures. It is simple, easy to perform, and gives immediate results. It can help in preventing subjective variations in determining the endpoint of treatment and can be used to assess early catheter removal after HJ stricture dilatation.
